# Effects of rTMS treatment on global cognitive function in Alzheimer's disease: A systematic review and meta-analysis

**DOI:** 10.3389/fnagi.2022.984708

**Published:** 2022-09-08

**Authors:** Tianjiao Zhang, Youxin Sui, Qian Lu, Xingjun Xu, Yi Zhu, Wenjun Dai, Ying Shen, Tong Wang

**Affiliations:** ^1^Rehabilitation Medicine Center, The First Affiliated Hospital of Nanjing Medical University, Nanjing, China; ^2^Department of Rehabilitation Medicine, The Affiliated Jiangsu Shengze Hospital of Nanjing Medical University, Suzhou, China

**Keywords:** Alzheimer's disease, repetitive transcranial magnetic stimulation (rTMS), cognitive function, MMSE, ADAS-cog, systematic review, meta-analysis

## Abstract

**Background:**

Although repetitive transcranial magnetic stimulation (rTMS) has been extensively studied in patients with Alzheimer's disease (AD), the clinical evidence remains inconsistent. The purpose of this meta-analysis was to evaluate the effects of rTMS on global cognitive function in patients with AD.

**Methods:**

An integrated literature search using 4 databases (PubMed, Web of Science, Embase, and Cochrane Library) was performed to identify English language articles published up to October 6, 2021. We pooled Mini-Mental State Examination (MMSE) and Alzheimer's Disease Assessment Scale-cognitive subscale (ADAS-Cog) scores using a random-effects model *via* RevMan 5.4 software. We calculated estimates of mean differences (MD) with 95% confidence intervals (CI). The primary outcomes were pre-post treatment changes in global cognition as measured using MMSE and ADAS-Cog immediately after rTMS treatment, and the secondary outcome was duration of cognitive improvement (1–1.5 and ≥3 months).

**Results:**

Nine studies with 361 patients were included in this meta-analysis. The results showed that rTMS significantly improved global cognitive function immediately following rTMS treatment [(MD) 1.82, 95% confidence interval (CI) 1.41–2.22, *p* < 0.00001, MMSE; 2.72, 95% CI, 1.77–3.67, *p* < 0.00001, ADAS-Cog], and the therapeutic effects persisted for an extended duration (2.20, 95% CI, 0.93–3.47, *p* =0.0007, MMSE; 1.96, 95% CI, 0.96–2.95, *p* = 0.0001, ADAS-Cog). Subgroup analyses showed that high frequency rTMS targeted to the left dorsolateral prefrontal cortex (DLPFC) for over 20 sessions induced the greatest cognitive improvement, with effects lasting for more than 1 month after the final treatment. There were no significant differences in dropout rate (*p* > 0.05) or adverse effect rate (*p* > 0.05) between the rTMS and control groups.

**Conclusions:**

Repetitive TMS is a potentially effective treatment for cognitive impairment in AD that is safe and can induce long-lasting effects. Our results also showed that ADAS-cog and MMSE differed in determination of global cognitive impairment.

**Systematic review registration:**

http://www.crd.york.ac.uk/PROSPERO, PROSPERO CRD42022315545.

## Introduction

Alzheimer's disease (AD)-associated dementia accounts for 60–80% of dementia cases, and is a significant health concern in the elderly (Alzheimer's, 2018). Cognitive impairments are the main clinical manifestations of AD, and affect patients and caregivers, and increase societal burden (Yin et al., 2021). Drugs such as donepezil, rivastigmine, and galantamine commonly used for treatment if AD do not impact long-term prognosis (Liao et al., 2015). As a result, non-pharmacological interventions have received increased attention.

Repetitive transcranial magnetic stimulation (rTMS) is a non-invasive brain stimulation technique that affects brain metabolism and neurological function by regulating cortical excitability (Kobayashi and Pascual-Leone, 2003). Stimulation parameters of rTMS are critical to treatment effects. Low frequency rTMS (≤1 Hz) inhibits cortical excitability, high frequency rTMS (≥5 Hz) induces cortical excitability (Dong et al., 2018). Repetitive TMS can also enhance cognition through stimulation of specific cortical areas (such as DLPFC) (Alvarez-Salvado et al., 2014). Many *in vivo* and *in vitro* studies have shown that rTMS cognition in AD, and may be a promising therapy (Vlachos et al., 2012; Lenz et al., 2015; Zhao et al., 2017; Chen et al., 2019; Jia et al., 2021).

Four meta-analyses have been performed on the beneficial effects of rTMS on cognitive function in AD (Liao et al., 2015; Dong et al., 2018; Lin et al., 2019; Wang et al., 2020). However, these articles varied in rTMS parameters (i.e., treatment sessions, stimulation site, etc.) and the therapeutic outcomes. Furthermore, none have evaluated the effects of rTMS on cognitive function during follow-up. Although rTMS has shown promise as a treatment for AD, rTMS parameters and treatment schemes that produce favorable therapeutic value require further development. This meta-analysis summarized studies of global cognitive function in patients with AD.

## Methods and materials

Our work adhered to the preferred reporting items for systematic reviews and meta-analyses (PRISMA) guidelines (Moher et al., 2010) and was registered in the PROSPERO database for systematic reviews (CRD 42022315545).

### Search strategy

PubMed, Web of Science, Embase, and Cochrane Library were searched for relevant studies published before October 6, 2021. We used the following key words: (“Alzheimer disease” OR “Alzheimer's disease” OR “Alzheimer dementia” OR “Alzheimer's dementia” OR “Alzheimer syndrome” OR “Alzheimer type dementia” OR “AD”) AND (“transcranial magnetic stimulation” OR “repetitive transcranial magnetic stimulation” OR “brain stimulation” OR “TMS” OR “rTMS”). We also searched for additional unpublished and in-progress trials from ClinicalTrials.gov, and searched the reference lists of identified articles for additional studies.

### Eligibility criteria and quality assessment

The inclusion criteria for the study were as follows: (1) rTMS was performed on patients with Alzheimer's disease; (2) global cognition was quantitatively determined using the Alzheimer's Disease Assessment Scale-cognitive subscale (ADAS-cog) or Mini-Mental State Examination (MMSE); (3) rTMS was conducted alone or in combination with cognitive training; (4) the active rTMS control group received sham rTMS, cognitive training, or other treatments; (5) mean cognition outcome score changes and standard deviation (SD) were accessible, or absolute original scale scores were available to calculate mean ± SD; and (6) randomized controlled studies. The exclusion criteria were as follows: (1) animal studies; (2) included patients with neurological disorders other than Alzheimer's disease; (3) reviews, letters, comments, unpublished reports, or meeting minutes; (4) duplicate articles; and (5) non-English articles.

Cochrane Collaboration was used by two statisticians independently to determine study quality. The evaluation criteria were: (a) sequence generation; (b) allocation concealment; (c) blinding; (d) approach for handling incomplete outcome data; (e) selective reporting; and (f) other potential bias. The risk of bias was rated as low, high, or uncertain.

### Data extraction

Data from the included studies were extracted and examined by two researchers (TZ, YXS, and QL). During screening and comparison, disagreements were resolved by consensus between the researchers or by a third specialized researcher. For each included article, we recorded the following information: basic characteristics of the included studies (author, year of publication, and study design), sample size, disease type, rTMS parameters (number of sessions, frequency, intensity, stimulation targets), global cognitive performance (ADAS-cog or MMSE) score, follow-up time, and adverse effects.

### Data analysis

We used Rev-Man 5.4 software (Review Manger of Cochrane Collaboration) for all data synthesis and analysis. Effect size was computed by mean differences (MD) with 95% confidence intervals (CIs). The degree of heterogeneity was considered significant if the I^2^ statistic was >50%. Based on the inherent clinical heterogeneity in our pooled studies, a random-effects models was used for more conservative outcomes. Planned subgroup analyses were used for rTMS frequency, target sites, treatment sessions, and concomitant cognitive training. For all statistical analyses, *P* < 0.05 was considered significant. When heterogeneity between studies was significant, sensitivity analyses were performed using the leave-one-out technique. Due to the low number of included studies, Egger's tests were used instead of funnel plots, and *p* < 0.05 indicated publication bias (Egger et al., 1997).

### Search and selection of studies

**Figure 1:**
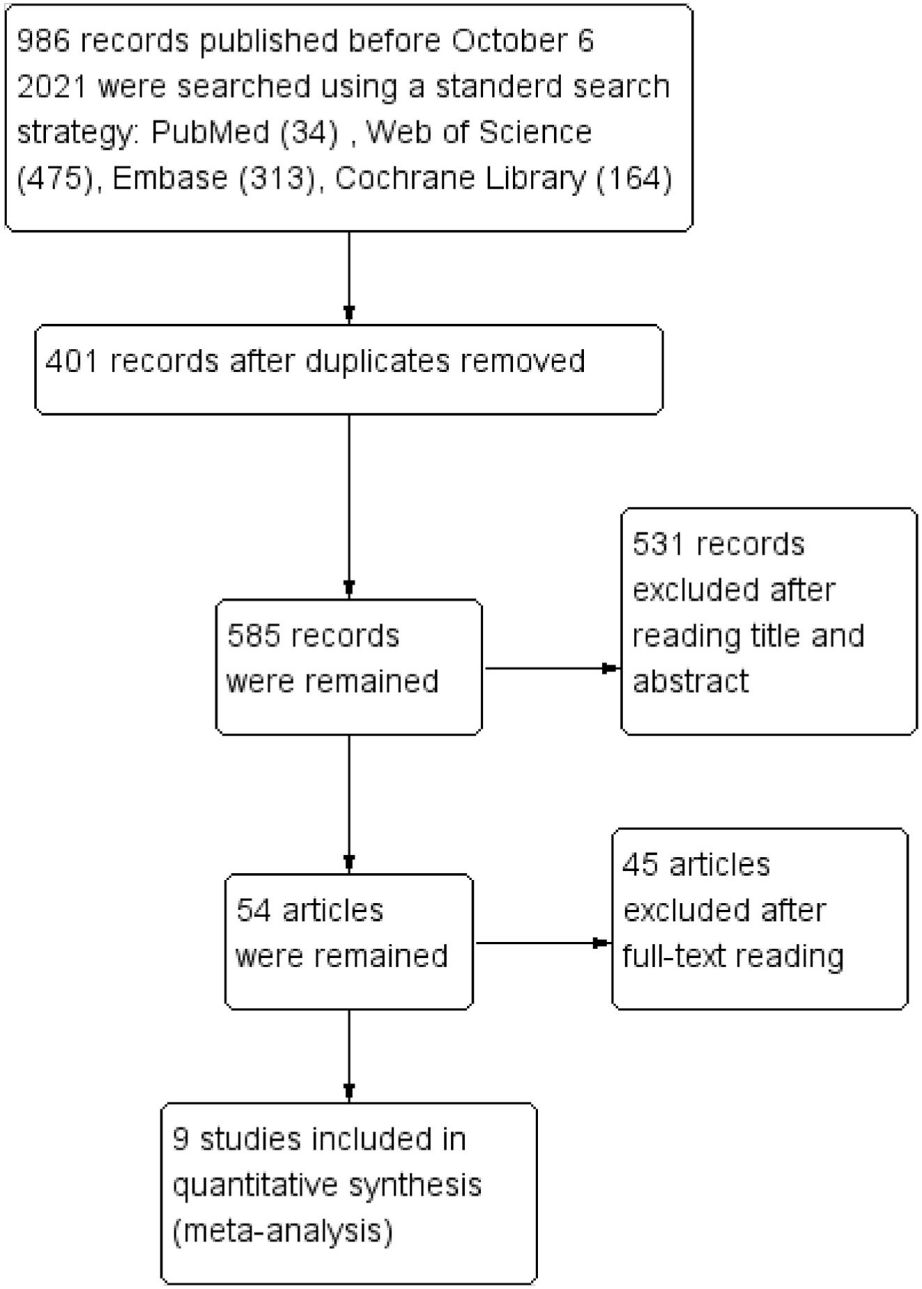
Flow diagram of study selection.

The search identified 986 studies from four English databases (PubMed, Web of Science, Embase, and Cochrane Library), and 401 duplicates were removed. After assessing titles and abstracts, 54 articles were include in the full-text reading phase, and nine studies were included in the meta-analysis. The study selection process is shown in Figure 1.

### Identifications and descriptions of the included studies

Supplementary Table 1 shows the characteristics of the nine trials included in this meta-analysis. The meta-analysis included 361 participants (192 in the rTMS group and 169 in the control group (Ahmed et al., 2012; Rabey et al., 2013; Wu et al., 2015; Lee et al., 2016; Zhao et al., 2017; Zhang et al., 2019; Brem et al., 2020; Jia et al., 2021; Li et al., 2021). Participants were diagnosed with AD based on different diagnostic criteria. However, one study reported the outcomes for mild to moderate AD and severe AD. Therefore, we chose the mild to moderate AD subgroup for the meta-analysis (Ahmed et al., 2012). For the interventions, four studies combined rTMS with cognitive training (Rabey et al., 2013; Lee et al., 2016; Zhang et al., 2019; Brem et al., 2020). Global cognitive function assessments included MMSE (six studies) (Ahmed et al., 2012; Lee et al., 2016; Zhao et al., 2017; Zhang et al., 2019; Jia et al., 2021; Li et al., 2021) and ADAS-Cog (seven studies) (Rabey et al., 2013; Wu et al., 2015; Lee et al., 2016; Zhao et al., 2017; Zhang et al., 2019; Brem et al., 2020; Li et al., 2021). Six studies assessed cognitive function at various follow-up times from 1 to 3 months after the final session (Ahmed et al., 2012; Lee et al., 2016; Zhao et al., 2017; Zhang et al., 2019; Brem et al., 2020; Li et al., 2021). Five trials reported adverse effects with diverse causes (Wu et al., 2015; Lee et al., 2016; Zhao et al., 2017; Zhang et al., 2019; Jia et al., 2021).

Supplementary Table 2 presents the characteristics of the rTMS protocols from the included studies. Only one study used low-frequency rTMS (Ahmed et al., 2012), and the other eight studies only used high-frequency rTMS. Five studies used a frequency of 10 Hz (Rabey et al., 2013; Lee et al., 2016; Zhang et al., 2019; Brem et al., 2020; Jia et al., 2021), and four studies used a frequency of 20 Hz (Ahmed et al., 2012; Wu et al., 2015; Zhao et al., 2017; Li et al., 2021). Repetitive TMS stimulation sites included DLPFC (Wu et al., 2015; Li et al., 2021), left lateral parietal cortex (Jia et al., 2021), bilateral DLPFC (Ahmed et al., 2012), left DLPFC, left lateral temporal lobe (LTL) (Zhang et al., 2019), parietal lobe, and posterior temporal lobe (Zhao et al., 2017). Three studies performed rTMS stimulation on the following six brain areas: (a) Broca's area, Wernicke's area, bilateral DLPFC and bilateral parietal somatosensory association (pSAC) (Rabey et al., 2013; Lee et al., 2016), (b) Broca's area, Wernicke's area, bilateral DLPFC, and bilateral inferior parietal lobule (Brem et al., 2020). The intensity of rTMS was 80–120% of resting motor threshold (rMT), and the number of interventions was 5–54 sessions.

### Research quality

**Figure 2:**
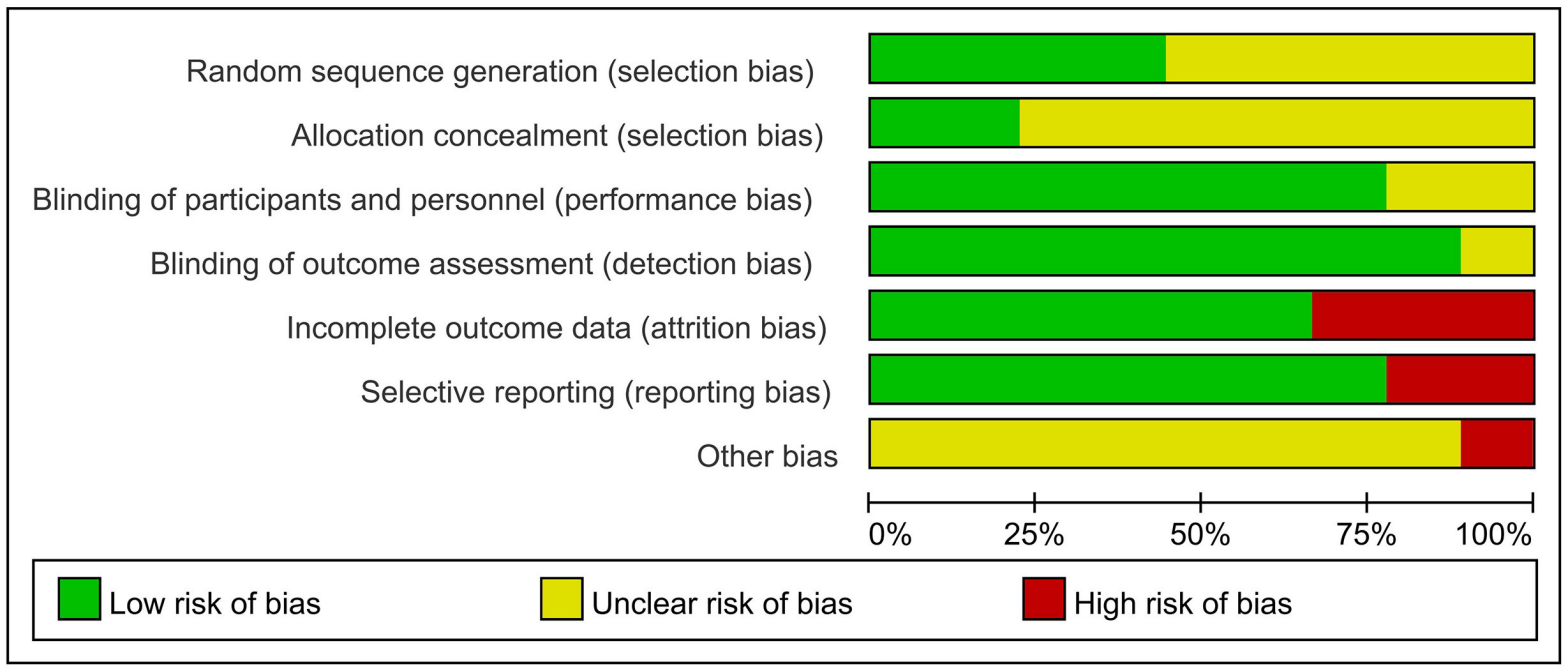
Risks of bias.

Figure 2 summarizes the risk of bias for the included studies. Each of the nine trials stated random allocation, but only four described how to generate the random sequence in detail and were rated as “low risk” (Wu et al., 2015; Zhang et al., 2019; Jia et al., 2021; Li et al., 2021). Only two studies described the allocation concealment procedure adequately (Brem et al., 2020; Jia et al., 2021). Seven trials reported that participants and researchers were double-blinded, and the remaining two trials did not mention blinding (Ahmed et al., 2012; Zhang et al., 2019). Outcome assessors were blind to group allocation in all studies except for one study; which did not indicate whether blinding occurred (Brem et al., 2020). The risk of attrition bias in three studies was rated as “high risk” because the research data were incomplete (Ahmed et al., 2012; Rabey et al., 2013; Brem et al., 2020). The reporting bias of two studies were rated as “high risk” owing to selectively reporting of pre-specified outcome indicators (Lee et al., 2016; Brem et al., 2020). Most studies with unclear information were rated as “unclear risk” in other potential sources of bias. However, center bias in one study resulted in “high risk” (Brem et al., 2020).

## Results

### Global cognitive function (immediately after the intervention)

**Figure 3:**
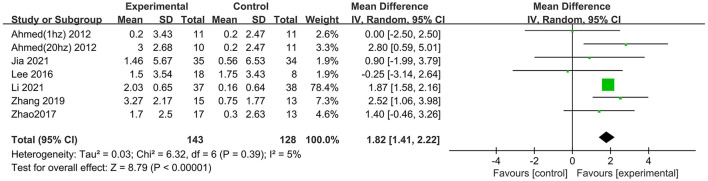
Forest plot of repetitive transcranial magnetic stimulation vs. the control group by MMSE immediately after the intervention.

**Figure 4:**
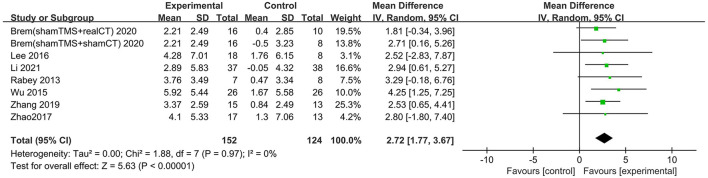
Forest plot of repetitive transcranial magnetic stimulation vs. the control group by ADAS-Cog immediately after the intervention.

Nine studies involving 361 participants with AD assessed the immediate post-treatment effect of rTMS on global cognitive ability. Six of the nine studies used MMSE (Ahmed et al., 2012; Lee et al., 2016; Zhao et al., 2017; Zhang et al., 2019; Jia et al., 2021; Li et al., 2021), and seven studies used ADAS-Cog (Rabey et al., 2013; Wu et al., 2015; Lee et al., 2016; Zhao et al., 2017; Zhang et al., 2019; Brem et al., 2020; Li et al., 2021). All global cognitive function results from MMSE and ADAS-Cog indicated that active rTMS treatment was superior to control treatment with a mean effect size of 1.82 (95% CI, 1.41–2.22, *p* < 0.00001, I^2^ = 5%, Figure 3) and 2.72 (95% CI, 1.77–3.67, *p* < 0.00001, I^2^ = 0%, Figure 4), respectively. Egger's regression showed no publication bias across studies that used ADAS-Cog (intercept = 0.16, df = 6, *t* = 0.08, two-tailed *p* = 0.94) or studies that used MMSE (intercept = −0.44, df = 5, *t* = 0.92, two-tailed *p* = 0.40). Sensitivity analysis was also performed, and omitting studies one by one did not alter the significance of the pooled MD.

### Subgroup analysis of global cognitive function (immediately after the intervention) by MMSE

**Figure 5:**
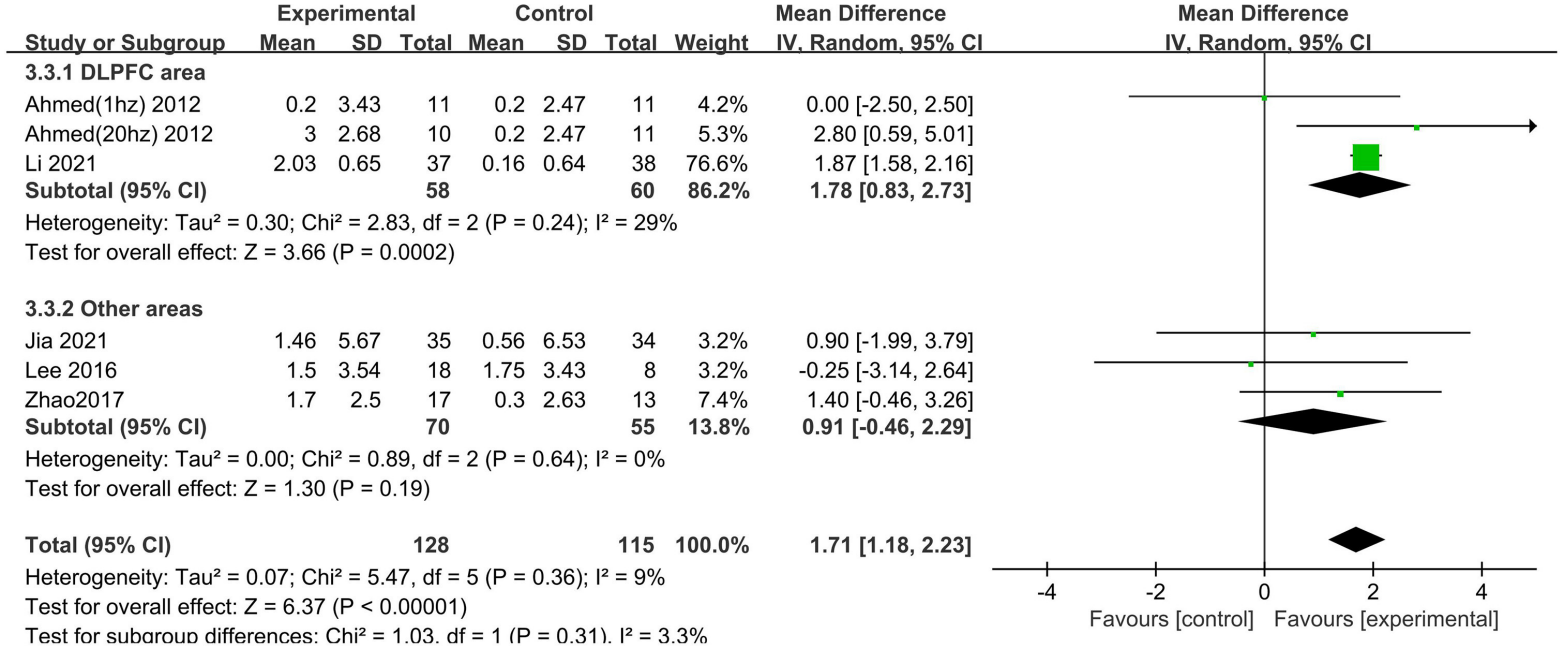
Subgroup analyses of repetitive transcranial magnetic stimulation vs. the control group by MMSE: DLPFC area vs. other areas.

Several subgroups were evaluated to identify variables that might influence the heterogeneity and cognitive outcomes by MMSE. Subgroup analysis was performed according to stimulation site. The efficacy of “Other areas” stimulation was 0.91 (95% CI −0.46 to 2.29), and a significant rTMS effect was found among those targeted to the DLPFC (MD = 1.78 95% CI 0.83– 2.73) (Figure 5). The excluded study by Zhang et al. included the left DLPFC and left lateral temporal lobe as the stimulus targets, so it could not be divided into either of the two subgroups.

**Figure 6:**
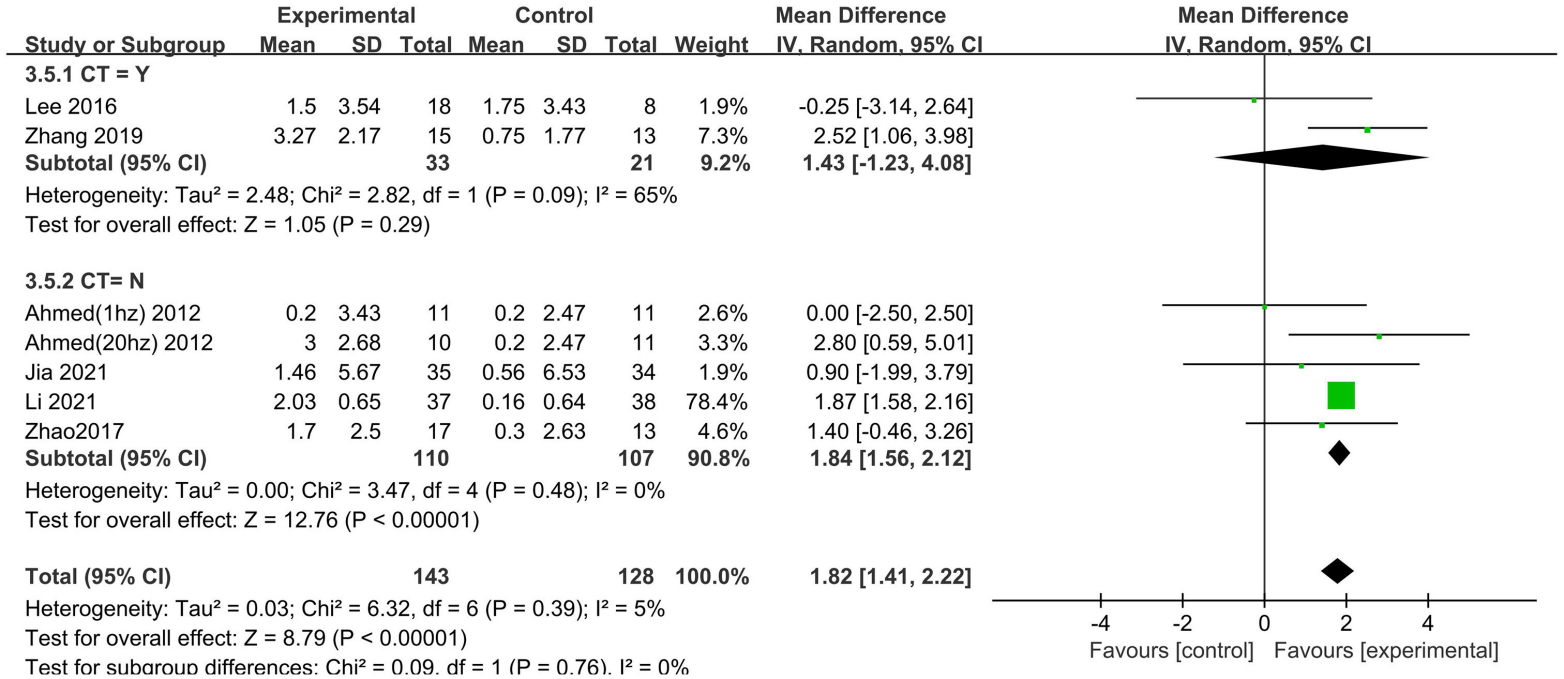
Subgroup analyses of repetitive transcranial magnetic stimulation vs. the control group by MMSE: CT = Yes vs. CT = No.

We also analyzed the effects of rTMS treatment in combination with cognitive training (“CT = Yes” vs. “CT = No”). A significant rTMS effect was found among studies that excluded cognitive training (1.84; 95% CI 1.56–2.12), but not in the studies involving cognitive training (1.43; 95% CI −1.23 to 4.08) (Figure 6).

**Figure 7:**
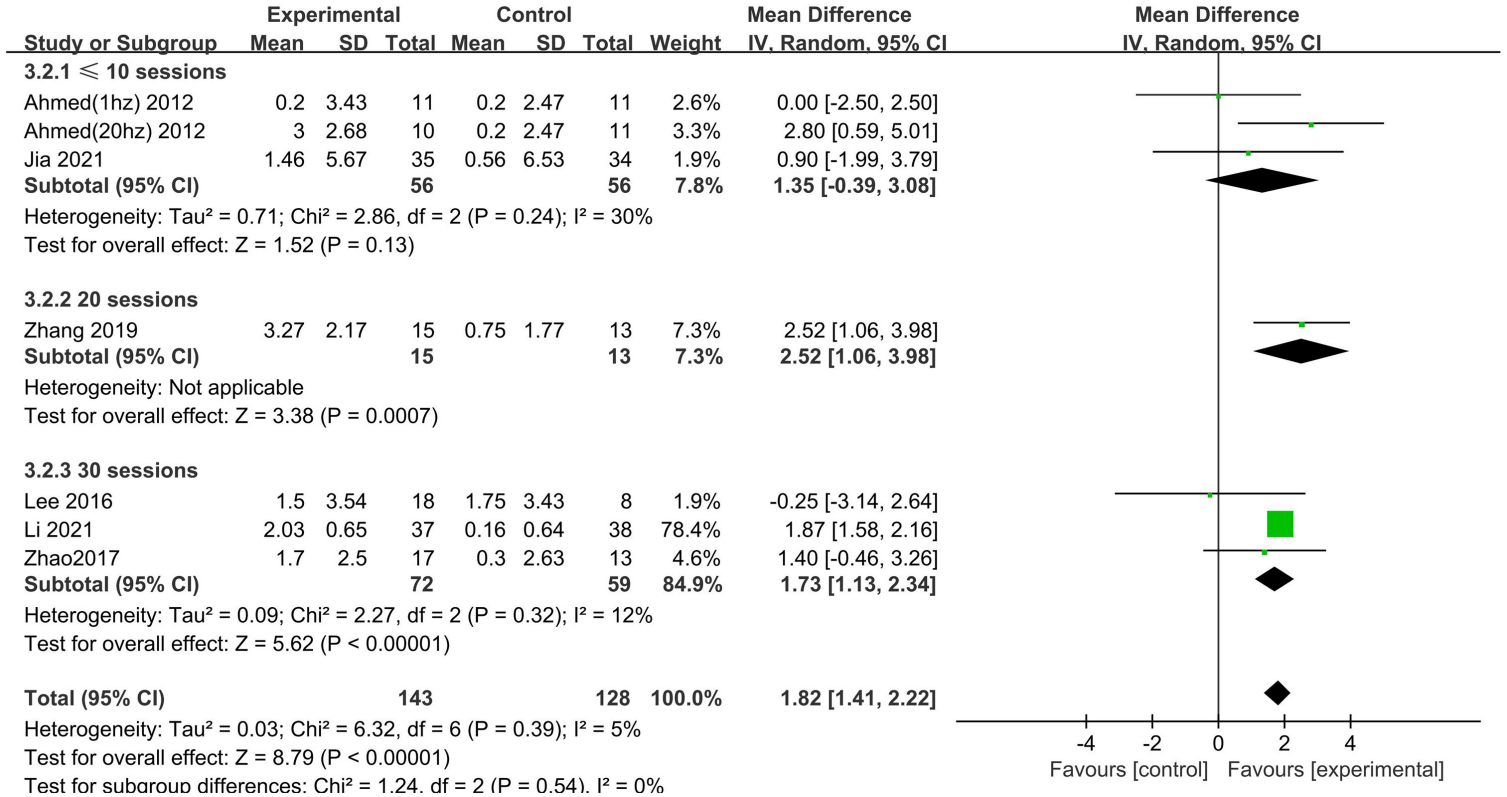
Subgroup analyses of repetitive transcranial magnetic stimulation vs. the control group by MMSE: ≤10 sessions vs. 20 sessions vs. 30 sessions.

Subgroup analysis for session number generated significant results for MMSE scores in the “20” and “30” subgroups (MD = 2.52 95% CI 1.06–3.98; MD = 1.73 95% CI 1.13–2.34), but not in the “ ≤10” subgroup (MD = 1.35 95% CI −0.39 to 3.08), which suggested that long-term rTMS treatment produced cognitive enhancement (Figure 7).

**Figure 8:**
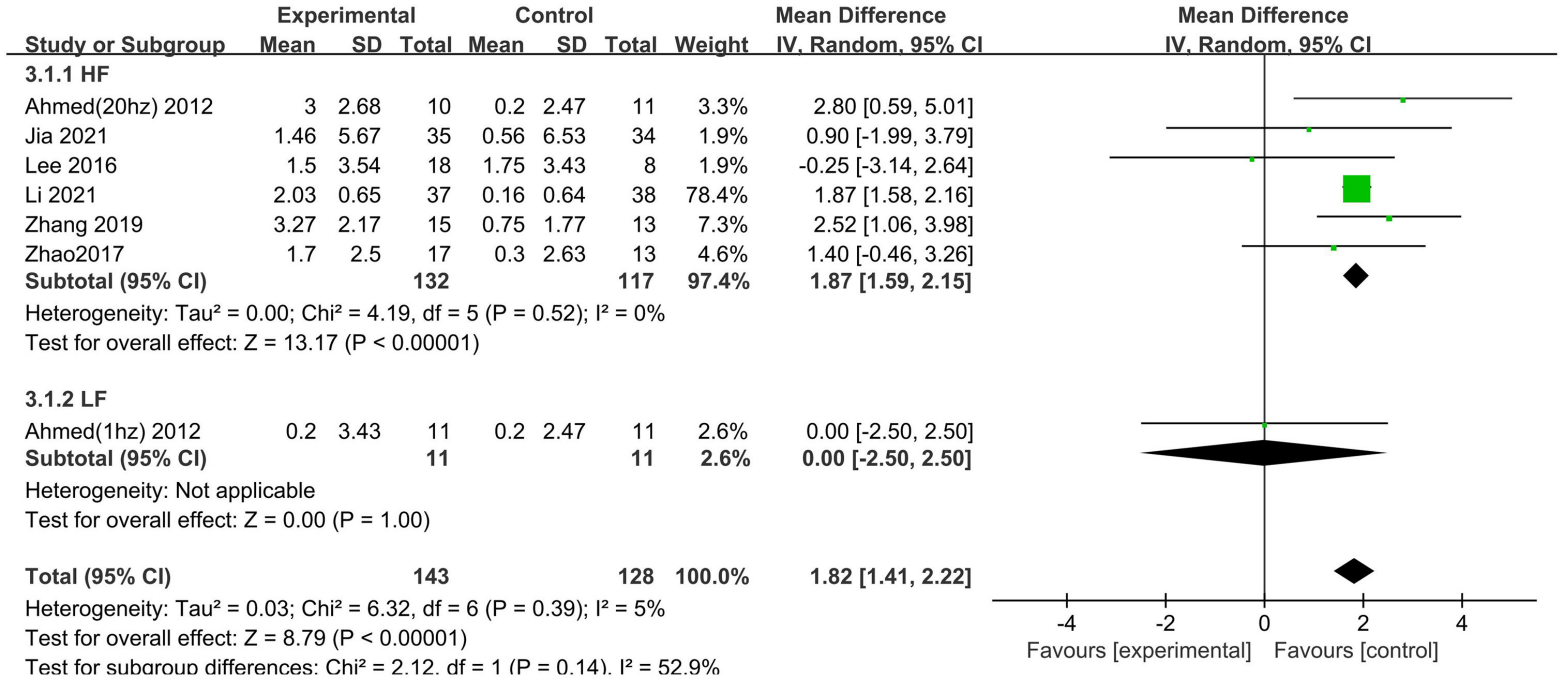
Subgroup analyses of repetitive transcranial magnetic stimulation vs. the control group by MMSE: HF vs. LF.

Treatment with high frequency stimulation result in a significant effect size of 1.87 (95% CI 1.59–2.15), whereas treatment with low frequency stimulation showed no positive effect 0.00 (95% CI −2.50 to 2.50) (Figure 8).

### Global cognitive function (follow-up)

**Figure 9:**
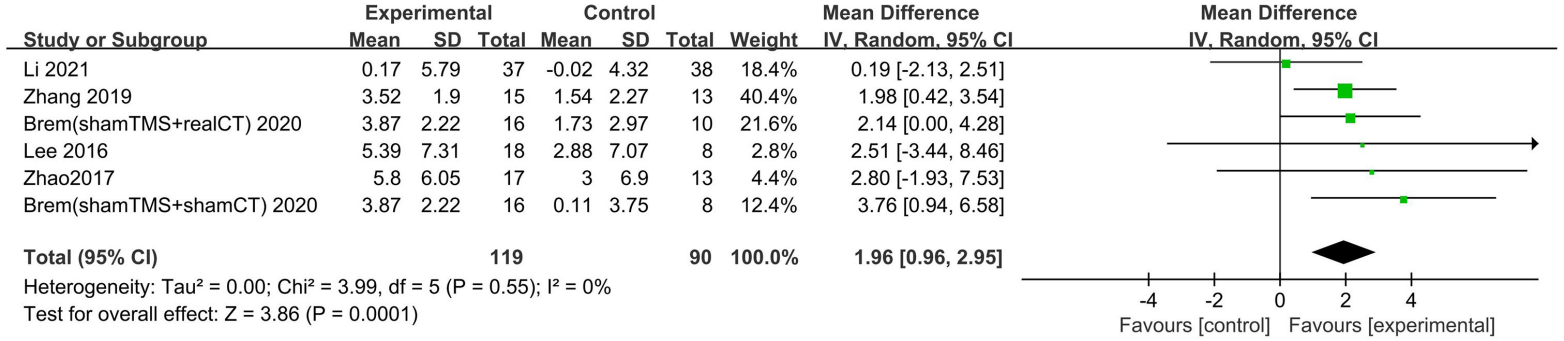
Forest plot of repetitive transcranial magnetic stimulation vs. the control group by ADAS-Cog at follow-up period.

**Figure 10:**
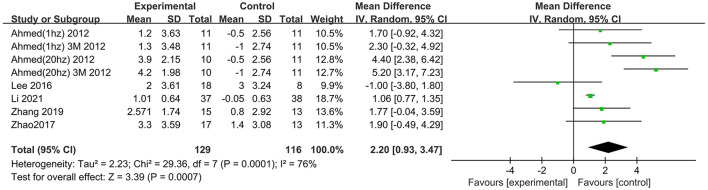
Forest plot of repetitive transcranial magnetic stimulation vs. the control group by MMSE at follow-up period.

Six trials assessed the effects of rTMS on global cognitive function at follow-up. The results showed low heterogeneity in the five studies that used ADAS-Cog (I^2^ = 0%, *p* = 0.55) (Lee et al., 2016; Zhao et al., 2017; Zhang et al., 2019; Brem et al., 2020; Li et al., 2021), The five studies that used MMSE had high heterogeneity (I^2^ = 76%, *p* = 0.0001) (Ahmed et al., 2012; Lee et al., 2016; Zhao et al., 2017; Zhang et al., 2019; Li et al., 2021). No significant asymmetry was found using Egger's regression test in studies that used ADAS-Cog (intercept = 0.37, df = 4, *t* = 0.32, two-tailed *p* = 0.76), and in MMSE (intercept = 1.59, df = 6, *t* = 2.28, two-tailed *p* = 0.06). The mean effect size was 1.96 for ADAS-Cog (95% CI, 0.96–2.95, *p* =0.0001, Figure 9) and 2.20 for MMSE (95% CI, 0.93–3.47, *p* = 0.0007, Figure 10). Both results showed that rTMS provided superior cognitive effects at follow-up compared with those in the control group.

### Subgroup analysis of global cognitive function (follow-up)

**Figure 11:**
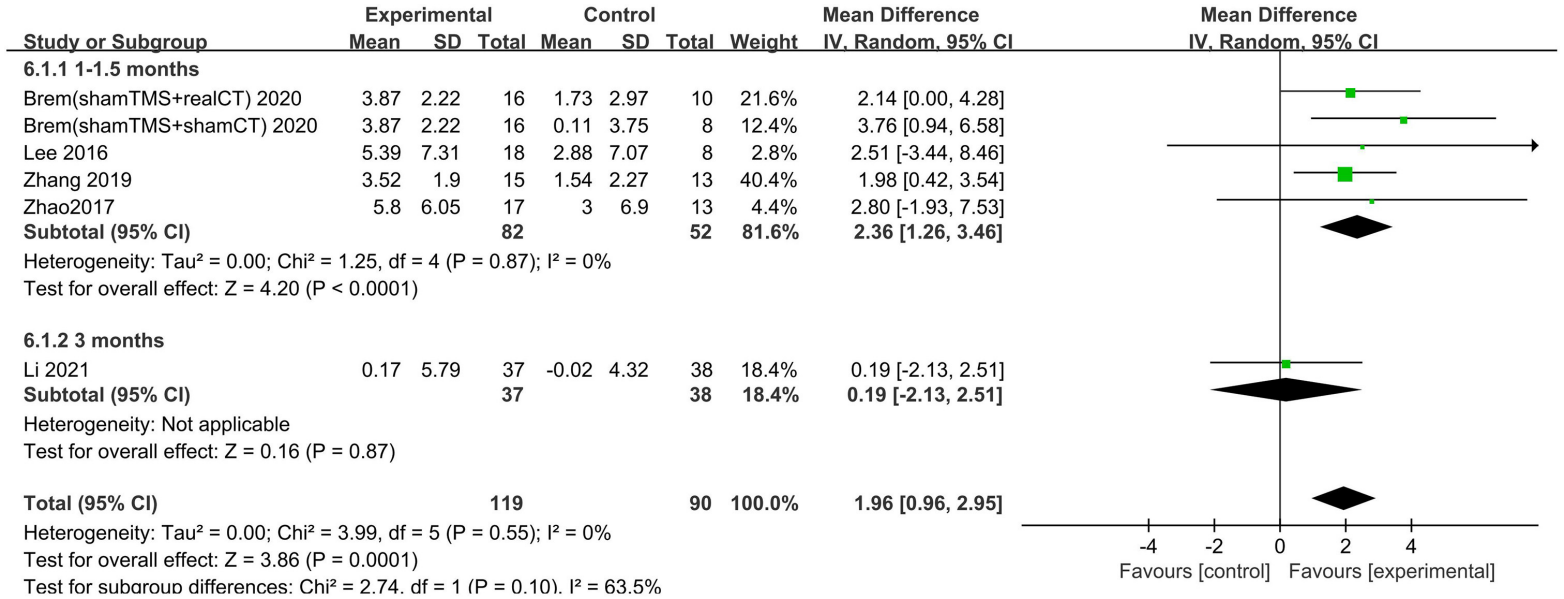
Subgroup analyses of repetitive transcranial magnetic stimulation vs. the control group by ADAS-Cog: 1–1.5 months vs. 3 months.

**Figure 12:**
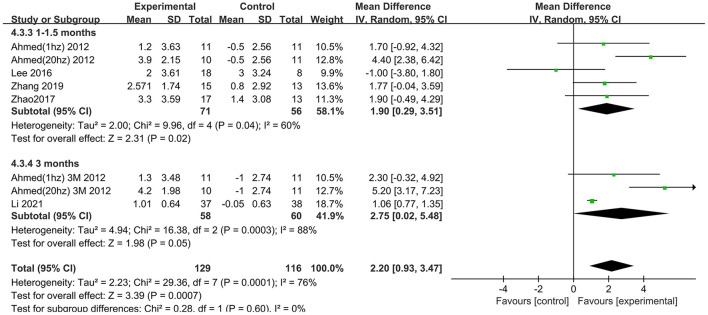
Subgroup analyses of repetitive transcranial magnetic stimulation vs. the control group by MMSE: 1–1.5 months vs. 3 months.

The follow-up times of the included studies ranged from 1 to 3 months. We divided the long-term effects into short-lasting effects (≤1.5 months) and longer-duration effects (3 months). The subgroup analysis of follow-up times revealed a mean effect size of 0.19 (95% CI −2.13 to 2.51) for ADAS-Cog with longer-duration effects. The mean effect size for short-lasting effects was 2.36 (95% CI 1.26–3.46). A significant rTMS effect was found at the shorter follow-up period (Figure 11). However, the results in two subgroup analyses (≤1.5 and 3 months) for MMSE were identical, with effect sizes of 1.90 (95% CI 0.29–3.51) and 2.75 (95% CI 0.02–5.48), respectively (Figure 12).

### Adverse effects and dropout

Of the 9 studies, 4 reported no adverse events during the study period and 3 reported adverse effects in both the active and sham groups (Wu et al., 2015; Zhao et al., 2017; Jia et al., 2021). Lee et al. described one patient in the sham arm who complained of mild headache and fatigue at the first follow-up visit (Lee et al., 2016). Seven participants who received rTMS in Zhang et al.'s (2019) study felt mild discomfort of the head, which eased after 3 days. Headaches, fatigue, and scalp discomfort were the most-reported adverse effects. The adverse effect rates were 12.6% (14/111) and 9.4% (10/94) in the active and sham groups, respectively, and the difference between the groups was not significant (χ^2^ = 0.192, *p* = 0.661).

Two studies did not report any dropout (Ahmed et al., 2012; Zhao et al., 2017). The drop-out rate was 10.5% (18/172) of the experimental group, with no significant difference compared with 14.1% (24/170) of the control group (χ^2^ = 1.059, *p* = 0.303).

## Discussion

Our review involved nine randomized sham controlled studies, and provided the most recent and detailed data regarding immediate and long-lasting treatment of global cognitive function in patients with AD following rTMS treatment. Subgroup analyses showed that rTMS protocols with high frequency and performed over the DLPFC for more than 20 sessions induced the best improvements in global cognitive function. In addition, we also showed that rTMS stimulation yielded cognitive benefits for 3 month in patients with AD assessed using MMSE, but not ADAS-Cog. Moreover, rTMS was safe and well-tolerated and did not induce severe adverse effects.

Consistent with a previous meta-analysis, high-frequency rTMS resulted in better therapeutic results than low-frequency rTMS for treatment of cognitive function. High-frequency rTMS altered synaptic plasticity and the level of brain-derived neurotrophic factor (BDNF). Studies showed a 55% reduction in synapses in early AD compared with mild cognitive impairment (MCI) and healthy subjects, as determined using electron microscopy, and the degree of synaptic loss correlated with cognitive deficits (DeKosky et al., 1996; Coleman and Yao, 2003). High-frequency rTMS attenuated synaptic damage in AD by inducing long-term potentiation (LTP) to increase synaptic activity, which is beneficial to learning and memory (Bliss and Collingridge, 1993). Memory formation is associated with BDNF, which is an important neurotrophic factor that promotes dendrite development and neuronal tolerance (Bekinschtein et al., 2008; Banerjee and Shenoy, 2021). A study showed that BDNF levels decreased with increased clinical severity of AD, and was most closely associated with memory decline in preclinical AD (Lim et al., 2021). One study reported that both high-frequency (20 Hz) and low-frequency (1 Hz) rTMS increased dopamine concentration and up-regulated the expression of dopamine receptor 4 in AD mice. However, only high-frequency rTMS intervention increased BDNF levels and enhanced the expression of Nestin and NeuN in brain tissue (Choung et al., 2021). In a review by Cheng et al., only two studies with relatively small sample sizes included low-frequency rTMS for treatment of patients with AD. Each of these studies showed superior efficacy of high-frequency rTMS on cognition (Ahmed et al., 2012; Ash et al., 2012; Cheng et al., 2018). These results should be treated with caution because few studies evaluated low-frequency rTMS.

Our meta-analysis found that stimulation of the DLPFC, the most common stimulation target for improving cognitive function in patients with AD, was most effective (Cotelli et al., 2011; Wu et al., 2015). Use of rTMS on the DLPFC improved cognition by directly stimulating this cortical region and activating connective circuits in distal structures (Dong et al., 2018). Our data differed from that of Alcalá-Lozano et al. (2018), which reported that rTMS over the left DLPFC and the six brain regions (Broca's area, Wernicke's area, bilateral DLPFC and bilateral pSAC) were similarly effective for improvement of cognitive function. Further studies are needed to determine the optimal targets for rTMS. In addition, our subgroup analysis of number of treatment sessions showed that long-term treatment (≥20 sessions) may have resulted in better cognitive improvement in patients with AD. Our findings agree with those of Lin et al. (2019), Jiang et al. (2020), Wang et al. (2020), and Xie et al. (2021).

Previous meta-analyses have reported significant effects (Sitzer et al., 2006; Cheng et al., 2018; Xie et al., 2021) and no effects (Lin et al., 2019; Chu et al., 2021) of combined CT with rTMS. In our study, the combination of rTMS and CT did not result in additional improvement. These results indicated that rTMS and CT may not have induced additive effects, and the combination may be counterproductive. Little is known about the underlying mechanisms by which rTMS and CT improve cognition. De Marco et al. suggested that cognitive training increased default mode network (DMN) connectivity in individuals with MCI, and a recent study noted that rTMS could induce deactivation of functional connectivity within the DMN to reduce cognitive deficits in patients with amnestic mild cognitive impairment (De Marco et al., 2018; Cui et al., 2019). Further studies are needed to explore the complexity of brain functional networks in patients with AD. However, Chu et al. indicated that CT may have been more effective for treatment of early AD than rTMS (Chu et al., 2021). Our results should be interpreted with caution since the subgroups had different sample sizes, and the CT designs were not consistent across the included studies.

The effective duration of rTMS in patients was also evaluated. In our meta-analysis, the effects of rTMS treatment lasted for 1–1.5 months, as determined using ADAS-Cog. Furthermore, outcomes were poorer with increased follow-up time. However, few studies have evaluated the duration of the effects of rTMS on cognition. Cotelli et al. (2011) showed persistent effects on sentence comprehension for 2 months with either 2 or 4 weeks of rTMS treatment applied over the left DLPFC. This finding agreed with the findings of another study that showed cognitive function improvement for 3 months after 5 days 20 Hz rTMS over the bilateral DLPFC (Ahmed et al., 2012). More data are needed to characterize the duration of the effects of rTMS treatment. Long-term effects may be related to modifications in functional connectivity of brain networks and synaptic plasticity in patients with AD patients through rTMS, because memory and learning activity can be regulated by synaptic neuronal activities (Demirtas-Tatlidede et al., 2013; Zhang et al., 2019). Furthermore, studies mainly reported off-line general cognitive improvement in early AD with less gray matter atrophy, which supported the role of network and synaptic impairment in patients with AD (Ahmed et al., 2012; Anderkova et al., 2015; Lee et al., 2016; Zhao et al., 2017). To determine the duration of rTMS efficacy and to determine the mechanisms underlying the effects of rTMS, future RCTs should include data on cortical excitability and functional magnetic resonance imaging.

## Limitations

This study was subject to the following limitations. First, we only focused on global cognitive ability of patients with AD, and it is possible we missed some Chinese literature due to the limited database searches we performed. Second, the optimal rTMS parameters are unclear because of small sample sizes and heterogeneous stimulation parameters of the included studies. Finally, because of the small number of included articles using both ADAS-cog and MMSE, consistent criteria for subgroup divisions for the two scales were difficult to determine. We only compared the efficacy of rTMS using ADAS-cog and MMSE at follow-up, which might have limited the ability to explore the differences between MMSE and ADAS-cog for evaluation of cognitive function. The results in the follow-up subgroup analysis showed that ADAS-Cog and MMSE differed in global cognitive function results in patients with AD, which led to the clinical evaluators having to choose the appropriate measure based on the intrinsic characteristics of the scales and the requirements for assessments.

## Conclusion

In conclusion, our meta-analysis showed that rTMS is a safe add-on therapy that induces cognitive enhancements in patients with AD. Patients with AD benefitted most from multiple high frequency rTMS sessions over the DLPFC, and post-treatment improvements persisted for at least 1 month. However, the optimal parameters of rTMS therapy could not be determined due to the limitations of the included studies. Additional investigations with large sample sizes and long-term follow-up are required to determine optimal rTMS parameters and to evaluate the long-term efficacy of rTMS for treatment of AD. Differences in ADAS-cog and MMSE scores also suggested that the clinical evaluator should choose the appropriate instrument based on the intrinsic characteristics of the scales and the requirements for assessments.

## Data availability statement

The original contributions presented in the study are included in the article/Supplementary material, further inquiries can be directed to the corresponding author/s.

## Author contributions

YS and TW conceived and propelled the study. TZ, YS, and QL screened literature and finished data extraction. XX and WD were in charge of risk of bias assessment and statistical analyses. TZ wrote and edited the manuscript. YZ offered guidance on meta methodology. All authors contributed to the article and approved the submitted version.

## Funding

This study was funded by the National Key R&D Program of China (Nos. 2018YFC2001600 and 2018YFC2001603) and the Nanjing Municipal Science and Technology Bureau (No. 2019060002).

## Conflict of interest

The authors declare that the research was conducted in the absence of any commercial or financial relationships that could be construed as a potential conflict of interest.

## Publisher's note

All claims expressed in this article are solely those of the authors and do not necessarily represent those of their affiliated organizations, or those of the publisher, the editors and the reviewers. Any product that may be evaluated in this article, or claim that may be made by its manufacturer, is not guaranteed or endorsed by the publisher.
