# Supplementary material for: Effects of rTMS treatment on global cognitive function in Alzheimer's disease: A systematic review and meta-analysis
Source: Front Aging Neurosci. 2022 Sep 8;14:984708. doi: 10.3389/fnagi.2022.984708 (PMC9492846; doi:10.3389/fnagi.2022.984708)
Supplement: Supplementary file 1 [file Table_1.DOCX]

Supplementary Material

Supplementary Table 1: Demographic characteristics of the nine included studies.

| **References** | **Study**  **design,**  **cognitive training** | **Interventions** | **AD type** | **Diagnostic criteria** | **Sample size** | **Follow-up** | **Outcome**  **measures** | **Adverse effects** |
| --- | --- | --- | --- | --- | --- | --- | --- | --- |
| Ahmed  2012 | Parallel,  no | HFrTMS  LFrTMS  Sham | Probable AD  (mild/moderate/  Severe) | NINCDS-ADRDA | 10  11  11 | 1 and 3 months | MMSE,  IADL,  GDS | No |
| Rabey 2013 | Parallel,  yes | HFrTMS+ CT  Sham+shamCT | Probable AD  (mild/moderate) | MMSE=18 to 24, CDR = 1, DSM-IV | 7  8 | No | ADAS-COG,  CGIC,NP | No |
| Wu  2015 | Parallel,  no | HFrTMS  Sham | Probable AD | MMSE<24,BEHAVE-AD>8,NINCDS-ADRAD | 26  26 | No | ADAS-COG,  BEHAVE-AD,TESS | Mild extrapyramidal  reactions,  headache |
| Lee  2016 | Parallel,  yes | HFrTMS+ CT  Sham+shamCT | Probable AD  (mild/moderate) | MMSE = 18 to 26,  CDR = 1 or 2, DSM-IV | 18  8 | 1.5 months | ADAS-COG, MMSE,  CGIC,GDS | Mild headache,  fatigability |
| Zhao  2017 | Parallel,  no | HFrTMS  Sham | AD  (mild/moderate) | MMSE = 18 to 26,  CDR = 1 or 2, DSM-IV | 17  13 | 1.5 months | ADAS-COG, MMSE,MOCA, WHO-UCLA AVLT | Mild headache, fatigue |
| Zhang 2019 | Parallel,  yes | HFrTMS+ CT  Sham + CT | Probable AD  (mild/moderate) | NINCDS-ADRDA | 15  13 | 1 months | ADAS-COG,  MMSE | Mild discomfort of the head, slight tingling in the scalp, mild muscle contraction around the area of stimulation |
| Brem  2020 | Parallel,  yes | HFrTMS + CT  Sham+CT Sham+shamCT | AD  (mild/moderate) | MMSE= 18 to 24,DSM 5, NIA-AA | 16  10  8 | 1 to 1.5 months | ADAS-COG, ADCS-CGIC, CDR | No |
| Li  2021 | Parallel,  no | HFrTMS  Sham | AD | MMSE=10 to 26, BEHAVE-AD>=8, DSM-IV | 37  38 | 3 months | ADAS-COG, MMSE | No |
| Jia  2021 | Parallel,  no | HFrTMS  Sham | Probable AD  (mild/moderate) | CDR =0.5-2, DSM-IV | 35  34 | No | MMSE,PVLT,CDR | Local scalp discomfort, fatigue |

*AD: Alzheimer’s disease; ADAS-Cog: Alzheimer’s Disease Assessment Scale-cognitive subscale; AVLT: Auditory Verbal Learning Test; BEHAVE-AD: Behavioral Pathology in Alzheimer’s disease rating scale; CDR: Clinical Dementia Rating; CGIC: Clinical Global Impression of Change; CT: cognitive training; DSM-IV: Diagnostic and Statistical Manual of Mental Disorders-IV; GDS: Geriatric Depression Scale; HFrTMS: high frequency rTMS; IADL: Instrumental Daily Living Activity; LFrTMS: low frequency rTMS; MMSE: Mini-Mental State Examination; MoCA: Montreal Cognitive Assessment; NIAAA: National Institute on Aging-Alzheimer's Association workgroups; NINCDS-ADRDA: National Institute of Neurological and Communicative Diseases and Stroke/Alzheimer’s Disease and related disorders association; NPI: Neuropsychiatric Inventory; rTMS: repetitive transcranial magnetic stimulation; TESS: Treatment Emergent Symptom Scale.
