# Supplementary material for: Effects of rTMS treatment on global cognitive function in Alzheimer's disease: A systematic review and meta-analysis
Source: Front Aging Neurosci. 2022 Sep 8;14:984708. doi: 10.3389/fnagi.2022.984708 (PMC9492846; doi:10.3389/fnagi.2022.984708)
Supplement: Supplementary file 2 [file Table_2.DOCX]

Supplementary Material

Supplementary Table 2: Descriptions of rTMS protocols in the included studies.

| **References** | **Stimulus**  **sites** | **Stimulus frequency & intensity & number of pulses each time** | **Treatment sessions** | **Treatment duration** |
| --- | --- | --- | --- | --- |
| Ahmed  2012 | bilateral DLPFC | 20Hz, 90%MT, 2000 pulses per session  1Hz, 100%MT, 2000 pulses per session | 5 sessions (1 day per week) | 5 days |
| Rabey 2013 | Broca, Wernicke,L/R DLPFC, L/R pSAC | 10 Hz, 90–110% RMT, 400 pulses for 2 brain sites and 500 pulses for 1 brain site per session | 54 sessions (5 sessions per week for 6 weeks and 2 sessions per week for 3 months) | 4.5 months |
| Wu  2015 | L-DLPFC | 20Hz, 80%RMT, 1200 pulses per session | 20 sessions (5 days per week) | 4 weeks |
| Lee  2016 | Broca, Wernicke, L/R DLPFC, L/R pSAC | 10 Hz, 90–110% RMT, 400 pulses for 3 brain sites per session | 30 sessions (5 days a week) | 6 weeks |
| Zhao  2017 | Parietal P3/P4 and posterior temporal T5/T6 | 20Hz | 30 sessions (5 days a week) | 6 weeks |
| Zhang 2019 | L-DLPFC and L-LTL | 10Hz, 100%MT, 1000 pulses per session | 20 sessions (5 days a week) | 4 weeks |
| Brem  2020 | Broca’s Wernicke’s areas, L/R DLPFC, L/R inferior parietal lobule | 10Hz, 120%MT | 30 sessions (5 days a week) | 6 weeks |
| Li  2021 | L-DLPFC | 20Hz, 100%MT, 2000 pulses per session | 30 sessions (5 days a week) | 6 weeks |
| Jia  2021 | left lateral parietal cortex | 10Hz, 100-110% MT, 800 pulses per session | 10 sessions (5 days a week) | 2 weeks |

*RMT, resting motor threshold; L, left; R, right; DLPFC, dorsolateral prefrontal cortex; LTL, lateral temporal lobe; pSAC, parietal somatosensory association
